# Novel Multiplex Near–Point-of-Care PCR Assay for Detecting Sexually Transmitted Infections Among PrEP Users in Western Kenya: Protocol for a Cross-Sectional Pilot Study With an Epidemiological Assessment

**DOI:** 10.2196/80643

**Published:** 2026-07-10

**Authors:** Aarman Sohaili, Felix Mogaka, Zoïe Alexiou, Victor Ocholla Omollo, Servaas A Morre, Elizabeth Anne Bukusi, Pierre P M Thomas

**Affiliations:** 1 Microbe & Lab BV, Matrix SIX, 1st Floor Sciencepark 408, Amsterdam, 1098 XH, The Netherlands, 31 616089665; 2 Department of Social Medicine, Care and Public Health Research Institute (CAPHRI), Faculty of Health, Medicine and Life Sciences, Maastricht University, Maastricht, The Netherlands; 3 Centre for Microbiology Research, Kenya Medical Research Institute, Kisumu, Kenya; 4 Department of Genetics and Cell Biology, Faculty of Health, Medicine and Life Sciences, Research Institute for Oncology and Reproduction, Maastricht University, Maastricht, The Netherlands; 5 Department of Medical Microbiology, Faculty of Health, Medicine & Life Sciences, Dutch Chlamydia trachomatis Reference Laboratory, Maastricht University, Maastricht, The Netherlands; 6 Department of Molecular and Cellular Engineering, Jacob Institute of Biotechnology and Bioengineering, Sam Higginbottom Institute of Agriculture, Allahabad, India

**Keywords:** STI, Kenya, diagnostics, point of care, chlamydia, gonorrhea

## Abstract

**Background:**

The global impact of sexually transmitted infections (STIs) significantly affects low- and middle-income countries (LMIC). Iin Kenya, where access to STI diagnostics is limited, effective diagnostic solutions are critically needed. Nucleic acid amplification tests are considered the laboratory gold standard for detecting pathogens such as *Chlamydia trachomatis* and *Neisseria gonorrhoeae* due to their high sensitivity and specificity. However, these methods typically require centralized laboratories, trained personnel, and longer turnaround times. FlashDx is a near–point-of-care molecular diagnostic platform designed to address these challenges by integrating automated sample processing and multiplex pathogen detection within a compact system suitable for decentralized use.

**Objective:**

The primary aim of this study is to validate the performance of the FlashDx STI multiplex assay for detecting STIs including *Chlamydia trachomatis*, *Neisseria gonorrhoeae*, *Trichomonas vaginalis*, *Mycoplasma genitalium*, *Mycoplasma hominis*, and *Ureaplasma* species compared with the reference Conformité Européenne In-Vitro Diagnostic (CE-IVD)-D dual real-time polymerase chain reaction (rtPCR) from Mikrogen.

**Methods:**

We propose a comparative cross-sectional study conducted with up to 400 young pre-exposure prophylaxis (PrEP) users aged between 15 years and 55 years at the Kenya Medical Research Institute Center for Microbiology Research Care and Training Program research site in Kisumu, Kenya. Urine samples were collected and analyzed using the FlashDx STI multiplex chip-based assay to detect 6 STIs, with results confirmed by the CE-IVD-D certified Mikrogen assay in the Netherlands. Participants were recruited in Kisumu, Kenya, from existing HIV prevention programs and sexual health services. Diagnostic performance will be assessed by calculating sensitivity, specificity, positive predictive value, and negative predictive value with 95% CIs. Descriptive and epidemiological analyses will summarize participant characteristics, behavioral risk factors, and the prevalence of STI infections within the cohort. Epidemiological data generated will include prevalence rates for all 6 STIs, providing an overview of STI prevalence with external validation to ensure accuracy and reliability.

**Results:**

The study was funded by Microbe & Lab, with in-kind contributions from the Kenya Medical Research Institute (KEMRI) Centre for Microbiology Research (CMR) Research Care and Training Program (RCTP). Data collection was conducted between April 2025 and June 2025 at the KEMRI research site in Kisumu, Kenya. A total of 400 participants were recruited for the study. The study is currently in the data analysis phase. Statistical evaluation of the diagnostic performance of the FlashDx STI multiplex assay is being carried out. In parallel, epidemiological analyses are being conducted. Final results and associated epidemiological findings are expected to be completed and prepared for publication in the summer of 2026.

**Conclusions:**

The findings from this study are expected to show the FlashDx STI multiplex assay as an effective point-of-care system for diagnosing 6 common STIs in settings such as Kenya. By demonstrating its usability, accuracy, and reliability, the FlashDx assay could be considered for broader implementation in clinical settings across Kenya and other LMIC.

## Introduction

The global burden of sexually transmitted infections (STIs) disproportionately affects low- and middle-income countries (LMIC), where the overlap with the HIV epidemic has been evident since its earliest days [1]. Bacterial STIs can lead to long-lasting complications such as pelvic inflammatory disease, chronic pelvic pain, tubal infertility, pregnancy complications, and increased susceptibility to HIV [1]. These physical, psychological, and social consequences of STIs are more severe and long-lasting among women [2].

*Chlamydia trachomatis* (CT) is a Gram-negative, obligate intracellular pathogen that causes a wide range of clinical presentations in humans [3]. CT infection can lead to trachoma, which is the leading cause of preventable blindness in the world, and CT is the most common bacterial STI globally [4]. In 2020, there were more than 129 million new cases of CT infections worldwide, a number that appears to be on the rise [5]. Although CT infections are treatable with antibiotics, they often remain asymptomatic, leading to delayed diagnosis and treatment [6]. Table 1 presents examples of CT prevalence measured among sexually active women in Kenya.

**Table 1.** *Chlamydia trachomatis* (CT) prevalence among sexually active women in Kenya.

Citation	Location	Study population (sample size)	Testing method	Samples tested	CT prevalence, %
Nyakambi et al (2021) [7]	Kisumu	SAF^a^ hospital attendees (n=385)	Rapid diagnostic test kit (Chinese commercial device)	Endocervical samples	7.5
Maina et al (2016) [8]	Nairobi	SAF at a family planning clinic (n=249)	NAAT^b^ (GenoQuick CT)	Endocervical samples	13
Nzioka (2016) [9]	Nairobi	SAF attending a reproductive clinic (n=197)	NAAT (Qiagen CT kit)	Endocervical samples	2
Lockhart et al (2019) [10]	Nairobi	FSWs^c^ attending a clinic for 2 years of follow-up (n=348)	NAAT (APTIMA COMBO2 assay)	Cervical samples	3.7
Yuh et al (2020) [11]	Nairobi	Girls with limited sexual experience (n=373)	NAAT (Gen-Probe APTIMA test)	Endocervical samples	11

a SAF: sexually active female.

b NAAT: nucleic acid amplification test.

c FSWs: female sex workers.

Gonorrhea, caused by the bacterium *Neisseria gonorrhoeae* (NG), poses a significant global health challenge, with an estimated 82 million new cases in 2020 [12]. This infection can present as urethritis in men and cervicitis or urethritis in women, with extragenital manifestations [13]. The World Health Organization (WHO) highlights the increasing incidence of gonorrhea due to asymptomatic cases and therapeutic failures, exacerbated by the bacterium’s ability to undergo high surface antigenic variation and develop antimicrobial resistance [14].

*Trichomonas vaginalis* (TV) is likely the most common nonviral STI globally and a significant public health concern due to its role in reproductive morbidity and facilitation of HIV transmission [15]. In women, TV can cause symptoms such as vaginitis and cervicitis, while infections in men are typically asymptomatic, contributing to its underdiagnosis and under-research. Growing evidence links TV infection to other high-morbidity diseases in both men and women, spurring increased efforts for diagnosis and treatment [16].

*Ureaplasma* species (US), *Mycoplasma genitalium* (MG), and *Mycoplasma hominis* (MH) are emerging urogenital pathogens associated with reproductive morbidity but are often underrecognized [17]. *Ureaplasma parvum* and *Ureaplasma urealyticum* are commonly present in the genital tract yet have been linked to adverse pregnancy outcomes, including preterm labor, premature rupture of membranes, chorioamnionitis, and neonatal infections [18]. MG is a sexually transmitted pathogen causing non-gonococcal urethritis in men and cervicitis and pelvic inflammatory disease in women, with largely asymptomatic infections contributing to ongoing transmission and antimicrobial resistance [19-21]. MH has also been associated with genitourinary and extragenital infections, pregnancy complications, and infertility [22]. Due to their fastidious growth and increasing antibiotic resistance, detection of these pathogens primarily relies on nucleic acid amplification tests (NAATs) [23].

Timely identification and accurate diagnosis of these STIs are critical, particularly in LMIC where STI epidemiology remains poorly understood. In these regions, political and sociocultural factors often deprioritize non-HIV STIs on public health agendas, resulting in inadequate surveillance and limited sexual health programs despite potentially significant disease burden [24]. The asymptomatic nature of these STIs underscores the need for evidence-based guidelines to implement targeted screening programs, especially among groups at high risk of STI acquisition [25-27].

Kenya, with an approximate population of 54 million people predominantly comprising those younger than 35 years, faces significant challenges regarding sexual and reproductive health [28]. There is a notable lack of awareness about STIs other than HIV [29]. Public health campaigns rarely address STIs or their link to reproductive issues such as infertility [30]. The prevalences of these STIs in Kenya remain understudied. This issue is compounded by the lack of insights on the strategies for diagnosis of these STIs in the Kenyan context [7].

There is a significant health burden of STIs in LMIC including Kenya. However, little progress has been made in these settings where syndromic STI management remains the standard of care due to high cost and lack of access to adequate diagnostic tools, including point-of-care (POC) platforms. Studies have found syndromic treatment to have a low diagnostic accuracy and to be less effective for vaginal syndrome than urethral syndromes [25]. Additionally, syndromic management cannot detect asymptomatic cases and more than three-quarters of women with an STI are asymptomatic [31]. Diagnostic tests to identify STI-causative organisms are widely used in high-income countries [26,32] However, these may not be feasible in LMIC, as diagnostic tests are largely unavailable [27]. Where testing is available, mostly in private health facilities, it is often expensive and geographically inaccessible, and patients often need to wait a long time (or need to return) to receive results [33].

NAATs are considered the gold standard for detecting a multitude of STIs due to their high sensitivity, high specificity, and rapid diagnostic capabilities in both symptomatic and asymptomatic cases [34]. However, NAAT testing is resource-intensive and costly and requires skilled technicians [33]. These limitations make traditional NAATs less practical in rural areas of Kenya [35]. Therefore, there is an urgent need for alternative diagnostic approaches. Current POC systems like the GeneXpert face challenges, including high costs of machinery and cartridges, infrastructure requirements, and limited 1-assay STI target inclusivity in their panels [36,37].

The multiplex near-POC FlashDx semi-Solid Phase Real-Time polymerase chain reaction (PCR; sSPRT) platform directly addresses the structural limitations observed in existing near-POC systems. Its higher multiplex capacity per cassette allows simultaneous detection of multiple pathogens within a single patient encounter, reducing repeat visits and missed co-infections [38]. Lower operational and per-test costs improve financial sustainability in high-volume HIV programs, while its compact and robust design reduces downtime risk and improves reliability in decentralized settings. The FlashDx platform itself is Conformité Européenne In-Vitro Diagnostic (CE-IVD) certified; however, the STI multiplex cassette used in this study is currently designated for research use only (RUO). The system is fully automated and delivers sample-to-result multiplex PCR in approximately 53 minutes, enabling rapid testing workflows that could support same-visit clinical decision-making and treatment initiation [32].

Therefore, in this study we intend to evaluate the performance and field usability of the FlashDx STI multiplex assay among pre-exposure prophylaxis (PrEP) users at an elevated risk of STI and HIV acquisition in Kisumu, Kenya. The assay enables simultaneous detection of 6 pathogens: CT, NG, TV, MG, MH, and US. The diagnostic performance of the RUO FlashDx assay will be compared with the CE-IVD–certified Mikrogen dual real-time PCR assay, which will serve as the laboratory reference standard. As the FlashDx platform is currently limited to research use, clinical decision-making during the study will rely on routine Cepheid GeneXpert testing for CT and NG, while FlashDx results will be externally validated through confirmatory testing at a reference laboratory in the Netherlands.

Beyond diagnostic validation, this study will generate epidemiological data on the prevalence of multiple STI pathogens among a high-risk population in western Kenya. Such data remain scarce but are essential for informing targeted public health interventions and improving STI control strategies. In addition, the study will assess the operational usability of the FlashDx system in field conditions, including ease of use, assay reliability, and implementation feasibility in decentralized health care settings.

Together, these findings will provide critical evidence on the potential of multiplex POC molecular diagnostics to expand STI detection in LMIC settings. It is hypothesized that the FlashDx multiplex assay will demonstrate effective diagnostic performance and will provide valuable epidemiological insights into the hidden prevalence and cocirculation of multiple STI pathogens among PrEP users in western Kenya.

## Methods

### Outcomes

The primary outcome of this study is the diagnostic performance of the FlashDx STI multiplex assay for the detection of the 6 STIs evaluated in comparison with the CE-IVD–certified Mikrogen PCR assays serving as the laboratory reference standard.

Diagnostic performance will be assessed using a predefined analytical checklist developed for this study to systematically evaluate assay accuracy. Results obtained with the FlashDx sSPRT STI panel will be compared with the Mikrogen ampliCube STD reference PCR assays. Samples yielding concordant results across both methods will be considered resolved. In cases where results are discordant, a third independent PCR assay will be performed, and the final infection classification will be assigned based on majority agreement, whereby a target will be considered positive only when at least 2 of the 3 assays produce a positive result. Diagnostic performance of the FlashDx platform will then be evaluated against this final resolved reference outcome. Sensitivity will be calculated as the proportion of true positives among all infected cases (true positives divided by the sum of true positives and false negatives), while specificity will be calculated as the proportion of true negatives among all noninfected cases (true negatives divided by the sum of true negatives and false positives). Positive predictive value and negative predictive value will also be calculated, along with corresponding confidence intervals using standard binomial methods.

Secondary outcomes include the generation of epidemiological data on the prevalence of multiple STIs within the study population, with external laboratory validation of results. In addition to CT and NG, the multiplex panel allows detection of US, MG, MH, and TV, providing a broader overview of STI distribution among PrEP users in western Kenya.

### Study Design

This study will use a comparative cross-sectional design aimed at validating the performance of the FlashDx STI multiplex assay for detecting STIs and estimating the prevalences of genital CT and NG.

### Study Setting

The study will be conducted in Kisumu, Kenya, focusing on PrEP users. Study activities will be carried out at the Kenya Medical Research Institute (KEMRI) Centre for Microbiology Research (CMR) Research Care and Training Program (RCTP) testing site located at Lumumba Sub-County Hospital. This site was selected due to its established infrastructure for HIV and STI research, its access to populations with a high burden of STIs, and its capacity to support molecular diagnostic testing using the in-house Cepheid GeneXpert.

### Study Population

The study enrolled 400 PrEP users aged 15 years to 55 years who may have been part of existing KEMRI PrEP cohorts or PrEP delivery points in Kisumu County. This specific cohort was selected to evaluate the effectiveness and applicability of the FlashDx STI multiplex assay in a population with a high burden of STIs who is already engaged in preventative health measures. The use of PrEP was an eligibility criterion for enrollment because individuals engaged in PrEP are considered a priority population for the prevention of curable STIs, given their high incidence, interest in longitudinal preventative services, and willingness to take pills for prevention. These participants will be retained in the study unless they choose to discontinue participation.

Eligible participants had to be willing and able to provide written informed consent and had to be confirmed as being HIV-seronegative according to the national HIV testing algorithm. Participants had to also be actively receiving PrEP at the time of enrollment. Recruitment occurred through pre-existing PrEP research cohorts at KEMRI-CMR-RCTP as well as through PrEP delivery clinics in the Kisumu area. Individuals were excluded if they had received prolonged antibiotic therapy, defined as more than a 14-day course within the month prior to enrollment, or if they had any active, clinically significant medical or psychiatric condition that, in the judgment of the site investigator or designee, could interfere with safe participation in the study.

### Power and Sample Size Determination

To accurately estimate the prevalence of STIs among sexually active women in Kisumu, Kenya, we based our calculations on an assumed CT prevalence of 10%, as indicated by collated studies from Sohaili et al [39] (range: 5%‐15%). The goal was to detect this prevalence with a precision of 5% (d) at a 99% confidence level (*z*=2.576).

To calculate the sample size, we used the Cochran formula for sample size estimation of a proportion:


$$n_{0}=\frac{Z^{2}\cdot p\cdot (1-p)}{e^{2}}$$

where *p* is the population size, *e* is the margin error, and *z* is the *z* value extracted from a *z* table.

### Sensitivity Analysis

To account for the variability in estimated prevalence (range: 5%‐15%), we performed a sensitivity analysis. For a prevalence of 5%, rounding up, the sample size would be approximately 127 participants. For a prevalence of 15%, rounding up, the sample size would be approximately 339 participants.

Based on this calculation, a minimum sample size of approximately 239 participants would be required to estimate STI prevalence with the specified precision. Sensitivity analyses using the lower and upper bounds of the expected prevalence range (5%‐15%) resulted in estimated sample sizes ranging from approximately 127 to 339 participants.

In addition, this study also aims to evaluate the diagnostic performance of the FlashDx assay relative to the reference PCR method. For diagnostic validation studies, larger sample sizes are desirable to ensure sufficient numbers of positive cases for reliable estimation of sensitivity, specificity, and agreement statistics. Assuming a CT prevalence of approximately 10%, a sample of 400 participants is expected to yield roughly 40 positive cases, which provides a reasonable basis for estimating diagnostic performance measures and conducting discrepancy analyses.

Therefore, a target sample size of 400 participants was selected to ensure adequate statistical power.

### Participant Recruitment

Participants were recruited using two complementary strategies. The primary approach involved a nested recruitment design in which eligible participants were invited from existing KEMRI cohorts enrolled in ongoing studies at the KEMRI-CMR-RCTP site. These participants were particularly suitable for inclusion because they already undergo routine screening for bacterial STIs such as CT and NG as part of established research protocols. Individuals who met the eligibility criteria were approached by study staff; provided with detailed study information, including a participant information sheet; and invited to provide written informed consent prior to enrollment.

If recruitment from existing KEMRI cohorts was insufficient or unavailable, participants were also recruited from PrEP delivery centers in Kisumu and surrounding areas. In this case, the experienced community outreach team at the KEMRI study site implemented community-based mobilization strategies to support recruitment. These efforts involved collaboration with community health volunteers, peer educators, youth peer providers, and other local gatekeepers to disseminate study information and encourage participation. Recruitment activities could include the distribution of printed and electronic information, education, and communication materials; engagement through social media platforms such as WhatsApp and Facebook; participation in community events for information sharing; and partnerships with local educational institutions to provide health talks and awareness sessions about the study.

Since recruitment occurred from both established research cohorts and community PrEP clinics, there is potential for selection differences between these groups. To address this, baseline demographic, behavioral, and clinical characteristics will be compared by recruitment source to assess potential selection bias.

Throughout the recruitment process, strict attention was given to protecting participant confidentiality and minimizing the risk of stigma. Potential participants were not approached individually in public or group settings, and recruitment messages were carefully reviewed to ensure cultural appropriateness and community acceptability. Individuals who declined participation or who did not meet the eligibility criteria were, where appropriate, provided with information about alternative studies or referred to standard HIV prevention and STI services available in the community.

Samples were tested within Kenya where feasible. The samples were also shipped to the Netherlands for further testing and validation of results. The testing procedures are described in the following sections.

### Sample Collection

Participants self-collected urine samples to test for STIs. Urine samples were chosen because they are suitable for STI testing using the FlashDx STI multiplex assay, the Cepheid GeneXpert, and the CE-IVD-D Mikrogen assay. The FlashDx system requires 120 μL of urine to run. The urine specimen collection occurred as follows. The study assistant labeled the urine collection container with the participant identification number and the date of sample collection before providing it to the participant. The participant details were then captured in the laboratory request form.

To obtain good specimans, participants were advised how to produce and collect a satisfactory urine sample in a cup using the following instructions: The cup is very clean. Do not open it until you are ready to use it. Provide first-catch urine (20-50 mL) in a urine collection cup free of any preservatives. Screw the cap on tightly so it does not leak. Put the cup into the box or bag that the study staff provided. Give the cup containing the urine sample to the staff.

The study staff stored the cup containing the urine sample in the cooler box containing ice packs ready for use in the laboratory.

### Sample Testing

All STI screening was completed with POC testing in Kisumu, Kenya, using the FlashDx STI panel. The urine samples were also sent to the Netherlands to be tested with the reference CE-IVD-D Mikrogen assay. These diagnostics are described in detail in the following sections.

#### FlashDx STI Panel

##### Overview

The FlashDx STI panel is designed for rapid molecular detection of 6 sexually transmitted pathogens associated with reproductive tract infections: CT, NG, TV, MG, MH, and US. The multiplex format allows simultaneous detection of multiple pathogens within a single assay, enabling identification of mixed infections and detection of asymptomatic cases.

The FlashDx platform itself is CE-IVDR certified; however, the STI multiplex cassette used in this study is currently designated as RUO and is therefore not approved for routine clinical diagnostics. The assay uses sSPRT technology and is performed using the FlashDx-1000-E system. The system integrates automated nucleic acid extraction, amplification, and detection, enabling sample-to-result molecular testing in approximately 53 minutes with minimal hands-on time.

The FlashDx system can process samples on demand without the need for batch testing, making it suitable for decentralized testing environments. The instrument is stored at 2 °C to 8 °C and includes an internal process control to ensure assay validity and quality control during each run.

##### Targets

Table 2 shows the targets that are used in this study for each sexually transmitted pathogen associated with reproductive tract infections.

**Table 2.** Targets for the sexually transmitted pathogens associated with reproductive tract infections.

Sexually transmitted pathogen	Target
*Chlamydia trachomatis*	Cryptic plasmid
*Ureaplasma* spp	ureC gene
*Mycoplasma hominis*	16S rRNA
*Neisseria gonorrhoeae*	Genomic target
*Mycoplasma genitalium*	mgpB gene
*Trichomonas vaginalis*	18S rRNA

##### Handling of FlashDx Results

Because the STI multiplex cassette used in this study is currently classified as RUO, individual FlashDx test results will not be returned to participants for clinical decision-making. Clinical management of participants will instead follow existing care pathways at the study site. Participants recruited from KEMRI PrEP cohorts routinely undergo STI testing for CT and NG using the Cepheid GeneXpert CT/NG assay as part of standard research protocols or clinical care.

All participants will also undergo routine clinical evaluation by a qualified medical professional who will assess symptoms and provide treatment according to national syndromic management guidelines where appropriate. Participants will be informed during the consent process that FlashDx testing is performed for research purposes only and that individual results from this assay will not be communicated to them.

### Sample Storage and Export to the Netherlands

Urine samples collected during the study were aliquoted and stored at −80 °C at the KEMRI research laboratory. Samples were subsequently exported to the Netherlands for confirmatory testing under a material transfer agreement and in accordance with both Kenyan and Dutch regulatory requirements governing biological sample transport and research use [40].

### CE-IVD Mikrogen Assay

The CE-IVD Mikrogen PCR assays are designed for various pathogen detections and are certified under the In-Vitro Diagnostics Directive 98/79/EC. These assays are known for their high sensitivity and specificity, providing reliable diagnostic results. These assays are compatible with most common real-time PCR cycler and various RNA/DNA extraction methods. The technology is CE-IVD certified.

**Figure 1.**
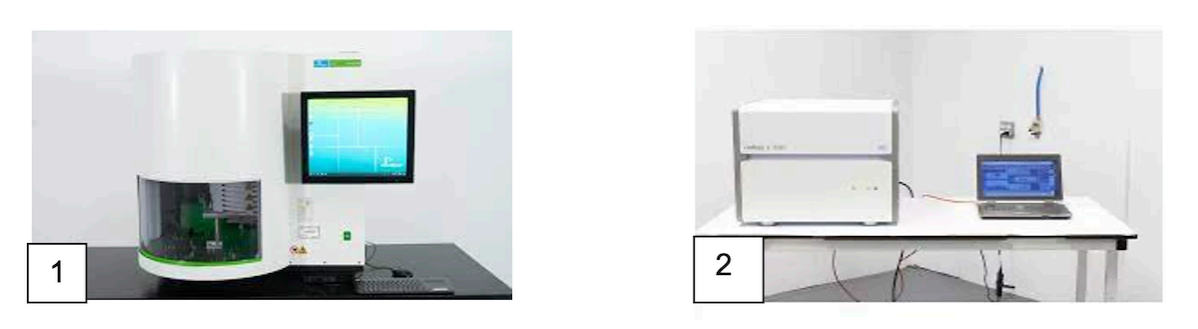
The Conformité Européenne In-Vitro Diagnostic (CE-IVD)-certified Mikrogen assay includes the following components: (1) Perkin elmer chemagic 360 nucleic acid extractor that uses chromagen magnetic bead technology for DNA isolation and (2) Roche LightCycler 480 for nucleic acid amplification.

For this study, the ampliCube STD panel 1 is used for real-time PCR detection of CT, NG*,* and MG. Moreover, the ampliCube STD Panel 2.1 is used for the detection of TV, MH, *Ureaplasma parvum*, and *Ureaplasma urealyticum* (Figure 1).

The diagnostic results obtained using the Mikrogen assay will in turn be compared with the results obtained using the FlashDX RUO tests. The presence of the different STIs will then be analyzed for alignment or discrepancy.

### Study Visits

Participants will attend the study site for 1 primary visit. This visit will be a screening visit where potential participants will provide informed consent and be assessed against inclusion and exclusion criteria. Once participants are confirmed as eligible, questionnaires will be administered to collect demographic and behavioral information. Following this, a 4-mL urine sample will be collected from each participant. Of this sample, 120 µL will be allocated for STI screening using the FlashDx Assay. If necessary, an additional 1 mL will be used for testing with the CEPHEID GeneXpert assay. The remaining sample will be stored and shipped to the Netherlands for further testing with the Mikrogen assay.

Participants may return to the study clinic for interim visits for any reason and will undergo STI testing and treatment if symptoms are present. As participants are part of PreP cohorts and may be a part of concurrent KEMRI trials, they will also undergo routine testing for bacterial STIs such as CT and NG.

Laboratory testing will be conducted by qualified staff, and STI diagnoses will be reviewed by the study team. All participants diagnosed with an STI will receive same-day treatment or be asked to return to the research clinic for treatment using WHO and Kenya standard therapies. A follow-up visit will be scheduled 2 weeks after completing STI treatment to conduct a test to determine if the infection was cured, ensuring the effectiveness of the treatment and resolution of the infection.

### Training of Local Staff

The FlashDx testing platform was integrated into the KEMRI Kisumu setting, with a trained researcher from the Netherlands assisting in its implementation and providing training to local staff on sample handling and testing procedures. The researcher transferred his knowledge of the system to local staff. Additionally, the study results will be shared with local policymakers and decision-makers in the field.

### STI Management

Participants diagnosed with CT or NG through routine clinical testing using the GeneXpert CT/NG system were contacted by a member of the study team and invited to return to the study clinic for treatment at a time convenient for them. Treatment is provided free of charge and follows Kenyan national and WHO guidelines [41]. Uncomplicated chlamydia infections are treated with a single oral dose of azithromycin, while gonorrhea infections are treated with a single intramuscular dose of ceftriaxone combined with a single oral dose of azithromycin. Participants with suspected complicated infections, such as pelvic inflammatory disease, receive extended treatment with ceftriaxone followed by a 2-week course of azithromycin and metronidazole as clinically indicated.

Participants treated for a bacterial STI were asked to return to the clinic approximately 2 weeks after treatment for a test-of-cure visit and encouraged to inform their partners to seek testing and treatment. Participants developing STI symptoms during the study may return for further evaluation and treatment. All treatments follow Kenyan Ministry of Health and WHO standard-of-care guidelines and are provided through the study clinic or affiliated health facilities.

### Data Collection

Self-collected urine samples are tested to collect data on STI organism-specific diagnosis.

Participants completed a research assistant-administered demographic, sexual, and behavioral questionnaire to collect the data shown in Table 3. The questionnaire was adapted from validated instruments used in previous KEMRI studies and hence in line with WHO recommendations.

**Table 3.** Data collection variables.

Variables	Description
Structural	
Socioeconomic status	Employment; income; presence of running water, electricity, metal roof, TV, or phone at home
Individual	
Demographic factors	Age; race, ethnicity, or tribe; educational level; time spent traveling for clinic visit
STI^a^ risk perception	What do you think is your risk of getting (giving) STIs from (to) your partner; previous STI history
Social harms	Physical, social, and economic social harms
Dynamic	
Relationship	Partnership status, social support, partner violence
Sexual behavior	Sex frequency, condom use, sexual power
Fertility intention	Number of children, current pregnancy, goal number of children, fertility intentions
STI stigma	Scale plus disclosure (eg, serodiscordant status) to family, friends, or religious leaders; adverse effects

a STI: sexually transmitted infection.

### Statistical Considerations

#### Data Analysis

The collected data will be analyzed using statistical software such as SPSS (IBM Corp) and R. The analysis will be conducted in several stages to meet the specific objectives and test the hypotheses.

#### Descriptive Statistics

Descriptive statistics will be used to summarize the demographic and clinical characteristics of the study participants. This will include measures such as means, medians, ranges, and standard deviations for continuous variables and frequencies and percentages for categorical variables.

#### Inferential Statistics

To determine the prevalence of each STI, proportions will be calculated along with 95% CIs. Chi-square tests will be used to examine associations between categorical variables (eg, the presence of STIs and demographic factors). Logistic regression analysis will be performed to identify factors independently associated with STI prevalence.

#### Multivariable Analysis

Multivariable logistic regression models will be developed to assess the relationship between multiple predictors and the presence of each STI. Adjusted odds ratios and 95% CIs will be reported to identify significant predictors while controlling for potential confounders.

#### Diagnostic Accuracy Analysis

Concordance, positive percent agreement, and negative percent agreement will be calculated for detecting NG and CT between the FlashDx and Mikrogen assays. Descriptive statistics will be performed and presented as numbers and percentages and as a Venn-diagram. Kappa values will be used to test for agreement between the 2 assays. Furthermore, we will calculate the positive and negative predictive values, sensitivity, and specificity for the FlashDx assay as compared with the reference Mikrogen assay. To further evaluate the performance of the FlashDx assay, we will measure diagnostic accuracy such as likelihood ratios and receiver operating characteristic curves. *P* values <.05 will be considered statistically significant. Chi-square and McNemar tests will be used to calculate the *P* values.

Missing data will be assessed for frequency and patterns prior to analysis. Where feasible, analyses will be conducted using complete case data; however, the extent of missingness will be reported, and sensitivity analyses may be performed if missing data exceed predefined thresholds to assess potential bias.

#### Software Use

SPSS will be used for data entry, descriptive statistics, and initial inferential analyses.

R will be used for more advanced statistical modeling and data visualization. The *tidyverse* package in R will facilitate data manipulation and visualization, while the *glm* function will be used for logistic regression analysis.

### Study Monitoring

The study team will monitor the progress and safety of the study through regular internal review and reporting. Monthly summary reports will track participant accrual, recruitment sources, and key baseline characteristics. In addition, quarterly reports will summarize pooled study data, including data completeness, specimen collection rates, and any reported adverse events. Participant safety will be reviewed on an ongoing basis, with particular attention to the relationship between any reported adverse events and study procedures. Monthly monitoring will also include reviews of premature study discontinuations, treatment discontinuations, and the reasons for withdrawal to ensure protocol adherence and study integrity.

Participants may be withdrawn from the study under several circumstances. These include a participant’s request to withdraw from the study at any time; determination by the investigator that the participant is at significant risk of noncompliance with the study protocol or that continued participation may pose potential harm; or a decision by oversight bodies such as the institutional review board (IRB), ethics committee, the study funder, or the Office for Human Research Protections.

### Ethical Considerations

#### IRB Review

This study involves human participants and received ethical approval from the KEMRI Scientific and Ethics Review Unit (SERU; approval number: KEMRI/SERU/CMR/P00291/5102). The protocol, site-specific informed consent forms, participant recruitment materials, and all study-related documents were reviewed and approved in accordance with KEMRI SERU requirements (SERU reference KEMRI/05/24). Any protocol amendments or modifications will also be submitted for review and approval prior to implementation.

#### Informed Consent

Written informed consent was obtained from all participants prior to enrollment and before any study-related procedures were conducted. Participants received detailed information about the study objectives, procedures, potential risks, and benefits and had the opportunity to ask questions before deciding whether to participate. Consent forms were translated into relevant local languages and verified through independent back-translation to ensure accuracy and comprehension. Participation in the study is entirely voluntary, and participants may decline participation or withdraw from the study at any time without penalty or loss of access to standard medical care. Participants were also informed that certain laboratory analyses performed as part of the study are for research purposes only and that individual results from RUO assays will not be returned. Copies of the signed informed consent forms were offered to participants for their records and securely stored in accordance with institutional and regulatory requirements.

For participants who are minors (aged 15‐17 years), all procedures follow Kenyan legal requirements and IRB-approved ethical guidelines governing research involving adolescents. Written informed consent was obtained from a parent or legal guardian, and assent was obtained from the minor participant prior to any study-related procedures. Study staff provided age-appropriate explanations of the study, and minors were informed of their right to decline participation or withdraw from the study at any time without penalty. Confidentiality protections are maintained in accordance with ethical guidelines and local regulations.

#### Privacy and Confidentiality

Each study site established a standard operating procedure for confidentiality protection. This ensures that all study records, including administrative and regulatory documentation, as well as participant-related documentation such as informed consent forms, locator forms, case report forms, and notes of all participant contacts, are stored securely.

Every effort is being made to protect participant privacy and confidentiality. Personal identifying information is retained at the Kisumu study site and not forwarded to other labs. All information is identified only by study ID number. The site adheres to a standard operating procedure for confidentiality protection, which includes input from study staff and community representatives to identify potential confidentiality issues and strategies to address them.

All study-related information is stored securely at the study sites. Participant information is kept in areas with limited access. Data collection, administrative forms, laboratory specimens, and other reports are identified only by study ID number. Records containing names or other personal identifiers, such as locator forms and informed consent forms, are stored separately from study records identified by study ID number. All local databases have password-protected access systems. Forms, lists, logbooks, appointment books, and any other listings linking participant ID numbers to identifying information are stored in a separate, locked file in an area with limited access.

#### Data Storage Locations

During the project, data are stored on secure KEMRI servers with encryption and strong access controls.

All study data and records will be retained in accordance with KEMRI and IRB requirements. Study data, including de-identified participant information, laboratory results, and study documentation, will be securely stored for a minimum period of 10 years after completion of the study and publication of the results.

#### Data Access

##### Authorized Personnel

Access to the data is restricted to authorized personnel only, including the team members. Nonanonymized data are handled exclusively by these individuals.

##### Access Control Measures

Data are stored on password-protected computers and servers. Any data shared between the chief investigator and the supervisor are transferred using encrypted data sharing methods to maintain security.

### Ensuring Data Security

#### Physical and Digital Security

Data are protected through both physical and digital security measures. These include encrypted databases, secure servers, and restricted physical access to data storage locations.

#### Minimizing Unauthorized Access

Strict access control protocols are in place, ensuring that only authorized personnel can access sensitive data. All data are stored on secure, password-protected devices, and encrypted data transfers are used for any data sharing.

### Risks

Participants may feel embarrassed or worried about STI and HIV testing conducted during the study. Anxiety or worry may also from providing urine samples, though the participants will do this in a self-collected way. Study questionnaires may make participants uncomfortable or embarrassed when discussing sexual behavior or partners. All study staff are trained to provide professional care in clinical sample collection and counseling. Counseling is available to participants who need it based on their study experiences.

Additional considerations apply to participants who are minors (aged 15‐17 years). Adolescents may face greater concerns related to privacy, stigma, or disclosure of sexual health information than adult participants. To minimize these risks, study procedures are conducted in private settings, and staff use age-appropriate communication to ensure that participants understand the study procedures and their rights. Confidentiality protections are strictly maintained in accordance with IRB-approved protocols and Kenyan ethical guidelines governing research involving adolescents. Counseling services are available to provide support and guidance for minors who may experience anxiety or emotional distress related to sexual health discussions or testing.

Study staff strive to protect participants’ privacy and confidentiality. However, participants’ involvement in the study could become known to others, potentially resulting in social harms such as unfair treatment, discrimination, or problems with family and community acceptance. Participants might face issues with their partners or experience intimate partner violence. Counseling and referrals are available if needed.

### Benefits

Participants receive free STI testing and treatment, which is not readily available in public clinics. Prompt treatment of STIs, including asymptomatic infections, can reduce the risk of pelvic pain, infertility, and other complications that untreated STIs can cause. Participants also receive clinical assessment and counseling related to sexual and reproductive health.

Minor participants (aged 15-17 years) may particularly benefit from access to confidential sexual health services, health education, and counseling provided by trained health care professionals. These services may help adolescents better understand STI prevention, safe sexual practices, and available health care resources while also facilitating early diagnosis and treatment where needed.

### Reimbursement

Participants are reimbursed for travel costs and compensated for time spent during study visits, in accordance with local guidelines and regulatory approvals. Each participant will receive 500 Kenyan shillings (US $3.86) for the study.

## Results

The study was funded by Microbe & Lab, with in-kind contributions from the KEMRI-CMR-RCTP. Participant recruitment and sample collection were completed between April 2025 and June 2025 at the KEMRI-CMR-RCTP study site in Kisumu, Kenya. The study timeline is shown in Table 4.

**Table 4.** Study timeline.

Study activities	0 months	Screening	1 month	3 months	6 months	9 months
Pre-study arrangements						
Protocol acceptance	✓	—^a^	—	—	—	—
Ethical approval	✓	—	—	—	—	—
Product registration	✓	—	—	—	—	—
FlashDx training	✓	—	—	—	—	—
Logistical arrangements	✓	✓	✓	✓	✓	✓
Study coordination						
Obtain informed consent	—	✓	—	—	—	—
Screen for inclusion and exclusion criteria	—	✓	—	—	—	—
Collect updated contact information	—	✓	✓	✓	✓	✓
Reimbursement	—	✓	✓	✓	✓	✓
Questionnaires						
Demographic information	—	✓	—	—	—	—
Behavioral questionnaire	—	✓	—	—	—	—
STI^b^ risk perception	—	✓	✓	✓	✓	✓
Social harms and IPV^c^	—	✓	✓	✓	✓	✓
Sexual behavior and exposure history	—	✓	✓	✓	✓	✓
PrEP^d^ adherence	—	✓	✓	✓	✓	✓
Fertility intention	—	✓	✓	✓	✓	✓
Quality of life	—	✓	✓	✓	✓	✓
Clinical study care						
Medication review	—	✓	✓	✓	✓	✓
General symptom assessment	—	✓	✓	✓	✓	✓
WHO^e^ pregnancy checklist	—	✓	✓	✓	✓	✓
STI symptom assessment	—	✓	✓	✓	✓	✓
Supply of condoms	—	✓	✓	✓	✓	✓
Contraception counseling and provision or referral	—	✓	✓	✓	✓	✓
Risk reduction counseling	—	✓	✓	✓	✓	✓
Sample collection^f^						
HIV testing (rapid test)	—	✓	✓	✓	✓	✓
Urine pregnancy testing (rapid test)	—	✓	✓	✓	✓	✓
Testing with FlashDx (urine samples)	—	—	✓	✓	—	—
CT^g^/NG^h^ testing confirmation with GeneXpert (from concurrent trials)	—	—	✓	✓	✓	—
Shipping of samples to the Netherlands	—	—	—	—	✓	✓
Sample testing with the CE-IVD^i^-D Mikrogen assay	—	—	—	—	✓	✓
Archive and analysis						
Results analyzed by study team	—	—	—	—	✓	✓
First drafts of manuscripts compiled	—	—	—	—	—	✓
Study published	—	—	—	—	—	✓

a Not applicable.

b STI: sexually transmitted infection.

c IPV: intimate partner violence.

d PrEP: pre-exposure prophylaxis.

e WHO: World Health Organization.

f Participants diagnosed with an STI will return 2 weeks following the treatment to test if the STI was cured.

g CT: *Chlamydia trachomatis*.

h NG: *Neisseria gonorrhoeae*.

i CE-IVD: Conformité Européenne In-Vitro Diagnostic.

**Figure 2.**
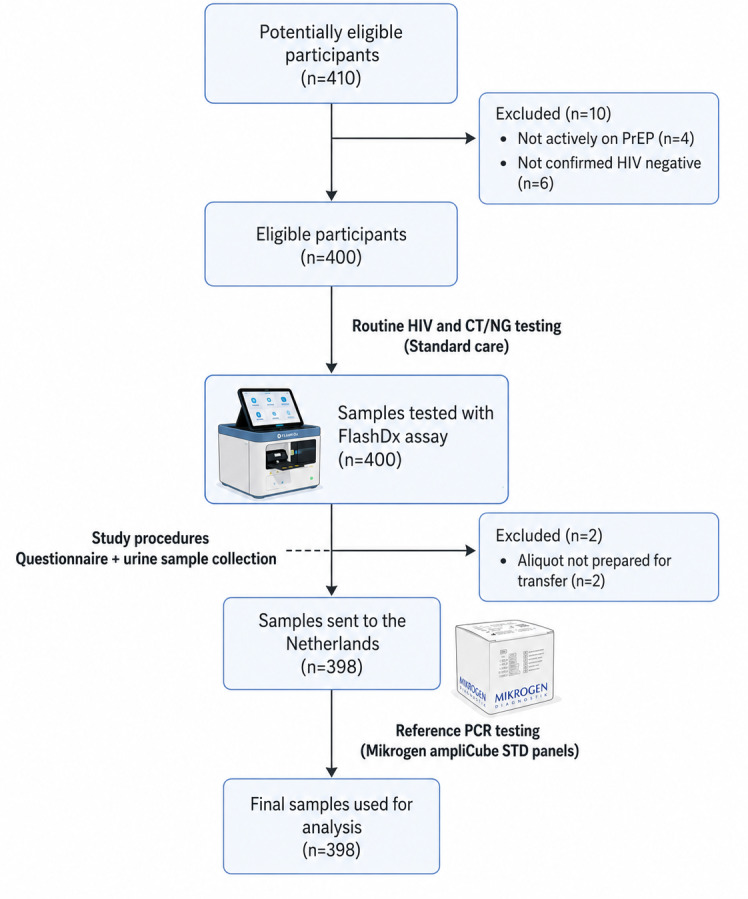
Study design, laboratory procedures, and STARD (Standards for Reporting of Diagnostic Accuracy Studies) participant flow for evaluation of the FlashDx sexually transmitted infection (STI) multiplex assay among pre-exposure prophylaxis (PrEP) users in Kisumu, Kenya. CT: *Chlamydia trachomatis*; NG: *Neisseria gonorrhoeae*; PCR: polymerase chain reaction; STD: sexually transmitted disease.

A total of 400 PrEP users meeting the study eligibility criteria were enrolled and provided urine samples for STI testing (Figure 2). Recruitment included participants from existing KEMRI PrEP cohorts as well as individuals attending PrEP delivery clinics in Kisumu and surrounding areas. Comparison of baseline demographic and clinical characteristics between participants recruited through cohort-based enrollment and those recruited through clinic-based pathways did not identify substantial differences between the groups.

All collected samples were initially processed using the FlashDx STI multiplex assay at the Kisumu testing site. Aliquots of the same specimens were stored and subsequently shipped to the Netherlands for confirmatory molecular testing using the CE-IVD–certified Mikrogen PCR assays. Laboratory processing of the confirmatory samples has been completed, and discrepancy analysis between the FlashDx assay and the reference Mikrogen assays is underway to assess diagnostic concordance and determine the overall diagnostic accuracy of the FlashDx platform.

In parallel, descriptive epidemiological data, including demographic characteristics, sexual health indicators, and preliminary information on the distribution of detected STI pathogens within the study population, have been compiled from the enrolled participants. Clinical follow-up procedures were successfully implemented, and a high proportion of participants diagnosed with bacterial STIs through routine clinical testing received appropriate treatment and follow-up care at the study clinic in accordance with Kenyan national guidelines.

Final results and associated epidemiological findings are expected to be completed and prepared for publication in the summer of 2026.

## Discussion

This study plans to evaluate the feasibility and diagnostic performance of a multiplex near-POC molecular platform for detecting STIs among PrEP users in western Kenya and provide an epidemiological profile of the population tested.

The anticipated principal finding is that the FlashDx multiplex assay will demonstrate good concordance with laboratory-based PCR methods while providing operational advantages that support decentralized STI testing. In addition to validating diagnostic performance, the study is expected to generate valuable epidemiological data describing the hidden prevalence and distribution of multiple STI pathogens within a high-risk population engaged in HIV prevention services.

These findings are particularly relevant in settings such as Kenya, where syndromic management remains the standard approach to STI treatment due to limited access to laboratory diagnostics [31]. Syndromic approaches have repeatedly been shown to miss asymptomatic infections and to have reduced diagnostic accuracy, particularly among women [27]. In contrast, molecular diagnostics used in high-income settings provide reliable pathogen detection but often require centralized laboratories, trained personnel, and extended turnaround times [32]. By evaluating a multiplex molecular platform that can operate in near-POC environments, this study addresses a critical gap between diagnostic performance and practical implementation in resource-limited settings.

The epidemiological component of this study is expected to contribute important data on the burden of STIs among PrEP users in western Kenya. Existing evidence suggests that the epidemiology of CT in Kenya remains incompletely characterized, with substantial heterogeneity between populations and geographic regions. A recent systematic review and meta-analysis of CT prevalence in Kenya [39], including 51 studies and more than 32,000 participants, reported a pooled CT prevalence of approximately 5.8% (95% CI 4.6‐7.4) across different populations, with considerable variation by sex, age, and risk group. The highest prevalence was observed among women in the general population older than 25 years, reaching approximately 14.8%.

Within this context, this study is expected to generate updated epidemiological data among PrEP users in Kisumu, a population already engaged in HIV prevention services but potentially at elevated risk for bacterial STIs. By simultaneously testing for multiple pathogens, this study may also provide insights into patterns of co-infection and asymptomatic infection that are rarely captured through routine surveillance systems.

The findings of this study will be disseminated to local, national, and international stakeholders through multiple channels to ensure that the results contribute to improved STI management, policy development, and scientific knowledge. The study will generate both quantitative and qualitative insights related to STIs, including epidemiological data, diagnostic performance outcomes, and operational findings relevant to STI testing strategies.

Dissemination materials will include summary reports, detailed technical analyses, policy-relevant recommendations, and supporting visual materials such as figures or infographics where appropriate. All shared information will comply with ethical and data protection requirements established by KEMRI and the National AIDS and STI Control Programme (NASCOP). To protect participant confidentiality, only de-identified or aggregated data will be shared externally.

At the local level, study findings will be communicated to health care providers and program stakeholders through presentations at the KEMRI Continuous Medical Education sessions held at Lumumba Sub-County Hospital. These sessions aim to support the translation of research findings into clinical practice within the local health system. Results will also be presented to the Kisumu County Health Management Team to support alignment with county-level health priorities and STI control initiatives. At the national level, comprehensive reports and policy briefs will be shared with the Ministry of Health through NASCOP to inform ongoing discussions related to STI diagnostic strategies and national guideline development.

Beyond local and national dissemination, study results will also be shared with the broader scientific community through publication in peer-reviewed journals and presentation at relevant regional and international conferences. These dissemination efforts aim to contribute to the global evidence base on STI epidemiology and diagnostic innovation in LMIC settings. Dissemination activities will be coordinated by the study leadership team in collaboration with health care professionals at KEMRI and relevant partners from NASCOP and the County Health Management Team.

Future research should explore the implementation of multiplex near-POC molecular diagnostics within routine sexual health services, particularly in high-burden settings where laboratory infrastructure is limited. Additional studies evaluating cost-effectiveness, integration within existing HIV programs, and long-term impacts on STI transmission and treatment outcomes would further inform policy decisions. The dissemination of findings to clinical providers, public health authorities, and national HIV and STI programs will also be important for translating study results into practice. Through presentations to local health authorities, policy briefings with national stakeholders, and peer-reviewed publications, the study aims to contribute evidence that may support improved STI diagnostic strategies in Kenya and other similar settings.

## Supplementary material

10.2196/80643 Checklist 1 SPIRIT (Standard Protocol Items: Recommendations for Interventional Trials) checklist.

## Abbreviations

CE-IVD: Conformité Européenne In-Vitro Diagnostic
CMR: Centre for Microbiology Research
CT: *Chlamydia trachomatis*
IRB: institutional review board
KEMRI: Kenya Medical Research Institute
LMIC: low- to middle-income countries
MG: *Mycoplasma genitalium*
MH: *Mycoplasma hominis*
NAAT: nucleic acid amplification test
NASCOP: National AIDS and STI Control
NG: *Neisseria gonorrhoeae*
PCR: polymerase chain reaction
POC: point of care
PrEP: pre-exposure prophylaxis
RCTP: Research Care and Training Program
RUO: research use only
SERU: Scientific and Ethics Review Unit
sSPRT: semi-Solid Phase Real-Time PCR
STI: sexually transmitted infection
TV: *Trichomonas vaginalis*
US: *Ureaplasma* species
WHO: World Health Organization
